# Innovative Hypofractionated Stereotactic Regimen Achieves Excellent Local Control with No Radiation Necrosis: Promising Results in the Management of Patients with Small Recurrent Inoperable GBM

**DOI:** 10.7759/cureus.536

**Published:** 2016-03-17

**Authors:** Angela Jia, Susan C. Pannullo, Shlomo Minkowitz, Shoshana Taube, Jenghwa Chang, Bhupesh Parashar, Paul Christos, A.Gabriella Wernicke

**Affiliations:** 1 Stich Radiation Oncology, NewYork-Presbyterian/Weill Cornell Medical Center; 2 Neurological Surgery, NewYork-Presbyterian/Weill Cornell Medical Center; 3 Radiology, NewYork-Presbyterian/Weill Cornell Medical Center; 4 Division of Biostatistics and Epidemiology, Department of Healthcare Policy and Research, NewYork-Presbyterian/Weill Cornell Medical Center

**Keywords:** gliomastoma multiforme, hypofractionated, stereotactic radiotherapy, small recurrent tumor

## Abstract

Management of recurrent glioblastoma multiforme (GBM) remains a challenge. Several institutions reported that a single fraction of ≥ 20 Gy for small tumor burden results in excellent local control; however, this is at the expense of a high incidence of radiation necrosis (RN). Therefore, we developed a hypofractionation pattern of 33 Gy/3 fractions, which is a radiobiological equivalent of 20 Gy, with the aim to lower the incidence of RN. We reviewed records of 21 patients with recurrent GBM treated with hypofractionated stereotactic radiation therapy (HFSRT) to their 22 respective lesions. Sixty Gy fractioned external beam radiotherapy was performed as first-line treatment. Median time from primary irradiation to HFSRT was 9.6 months (range: 3.1 – 68.1 months). In HFSRT, a median dose of 33 Gy in 11 Gy fractions was delivered to the 80% isodose line that encompassed the target volume. The median tumor volume was 1.07 cm3 (range: 0.11 – 16.64 cm3). The median follow-up time after HFSRT was 9.3 months (range: 1.7 – 33.6 months). Twenty-one of 23 lesions treated (91.3%) achieved local control while 2/23 (8.7%) progressed. Median time to progression outside of the treated site was 5.2 months (range: 2.2 – 9.6 months). Progression was treated with salvage chemotherapy. Five of 21 patients (23.8%) were alive at the end of this follow-up; two patients remain disease-free. The remaining 16/21 patients (76.2%) died of disease. Treatment was well tolerated by all patients with no acute CTC/RTOG > Grade 2. There was 0% incidence of RN. A prospective trial will be underway to validate these promising results.

## Introduction

Glioblastoma multiforme (GBM) is the most common primary brain malignant tumor in adults, with 22,850 new cases predicted in 2015 in the US [1]. Surgical resection alone results in a median survival of six months, which can be extended to 14.6 months with aggressive multimodality management [2]. In general, a higher radiation dose leads to increased survival [3]; however, conventionally fractionated doses of 2.0 Gy per fraction greater than a 60 Gy total dose provide no additional benefit while incurring a substantial increase in adverse effects [4-5]. As a diffusely infiltrative disease, local recurrence remains the most common form of failure pattern after standard treatment [6-7].

Following surgery, first-line adjuvant treatments include radiotherapy in combination with chemotherapy, often temozolomide (TMZ). Unfortunately, disease progression occurs in the majority of patients after a median progression-free survival of seven to 10 months [8]. Salvage systemic therapy include bevacizumab, a temozolomide rechallenge, carmustine, and carboplatin; however, no one agent has shown substantial survival benefits [9]. Localized treatment options for recurrent disease include surgery, stereotactic radiosurgery, hypofractionated stereotactic radiotherapy (HFSRT), and brachytherapy. Of these, HFSRT has emerged as a noninvasive method to deliver a high dose of radiation to the tumor site. In the treatment of recurrent GBM, numerous studies have documented a survival advantage on the use of HFSRT [10-18] and HFSRT in combination with chemotherapy [19-24]. Specifically, Iuchi, et al. [25] has shown that a single dose of more than 20 Gy is able to achieve durable local control in 72% of patients; however, radiation necrosis (RN) was observed in 43% of patients.

We hypothesized that a hypofractionation pattern of 33 Gy/3 fractions, which is a radiobiological equivalent of 20 Gy, could maintain therapeutic local control while reducing the incidence of RN. The present retrospective report evaluates the efficacy and safety of HFSRT in patients with small volume recurrent GBM.

## Materials and methods

### Patient selection

After IRB approval, we performed a retrospective analysis of 21 patients who had undergone treatment with BrainLab's Novalis Classic LINAC with 6-MV photons – based between 2011 and 2015. The inclusion criteria for the study was as follows: (1) histological confirmation of GBM; (2) a maximum diameter of recurrent tumor on magnetic resonance imaging (MRI) (gadolinium-enhanced T1 sequence) < 2.5 cm; and (3) Karnofsky Performance Scale (KPS) scores > 50. Informed patient consent was obtained at the time of treatment.

Initial treatments for all patients included a maximally safe neurosurgical resection followed by standard fractionation external-beam RT (median dose: 60.0 Gy in daily 2.0 Gy fractions) and concomitant temozolomide, as per the European Organisation for Research and Treatment of Cancer (EORTC) guidelines [26]. All patients were given maintenance TMZ. Tumor recurrence was confirmed on biopsy in 14 patients; in the other seven patients, recurrence was confirmed by diffusion-weighted MRI apparent diffusion coefficient (ADC) mapping and brain positron emission tomography (PET). Following tumor recurrence, salvage chemotherapy regimens were tailored to each patient and given with concurrent HFSRT; agents included bevacizumab, TMZ, and erlotinib.

### Radiation technique

The tumor was defined using Gd-enhanced MRI (Siemens Sonata, Siemens Medical Systems, Malvern, PA) with a stereotactic protocol using 3-mm slices for treatment planning purposes before the delivery of radiation. The MR image was co-registered and fused with CT scanning data (General Electric Medical System, U.K.). The clinical tumor volume (CTV) was designated to encompass the confines of the contrast-enhancing lesion. A uniform 3-mm expansion of the CTV was performed to create the planning target volume (PTV). Doses were prescribed to ensure at least 95% coverage of the PTV with the prescription dose. All gross disease was treated with HFSRT. Treatments were delivered using dynamic conformal arc radiotherapy. Patient immobilization was achieved using a commercially available head mask fixation system (BrainLab AG, Germany). Treatments were delivered with the BrainLab Novalis Classic LINAC with 6-MV photons. Imaging was provided with the BrainLab ExacTrac® positioning system. Irradiation was delivered in 3 fractions over three consecutive days to total dose of 33 Gy in 11-Gy fractions to the 80% isodose line that encompassed the target volume. For comparison of single and multiple dose regimens, the radiation dose was converted to a biological equivalent dose (BED) [27] and a single fraction equivalent dose (SFED) [28] according to the formulas below, assuming an α/β ratio of 8.6 for tumor control, and where D is total dose, d is dose per fraction, and D_q_ is 1.8:

$BED_{8.6}=D\times (1+\frac{d}{\alpha /\beta })$

$SFED=D-(n-1)\times D_{q}$

### Follow-up

Patients were clinically assessed every two months with physical examination and imaging. Specific attention was paid to neurological deficits and clinical symptoms of mass effect secondary to RN, such as seizures, headaches, personality changes, and motor or sensory deficits. Toxicities were graded according to the Radiation Therapy Oncology Group (RTOG) scale [29]. The use of dexamethasone after HFSRT was also noted.

Tumor status was monitored with a subsequent MRI every two months, or more frequently if symptomatic, utilizing the following sequences: T1-weighted, FLAIR, T2-weighted, gradient recalled echo, and diffusion-weighted imaging. Post-contrast Gd-enhanced T1-weighted MR images were obtained in the axial, sagittal, and coronal planes with 3-mm slice thicknesses. The size of the enhancing lesion was measured as the product of the largest cross-sectional diameter and the largest perpendicular diameter, in accordance to the Response Assessment in Neuro-Oncology Criteria (RANO) criteria [30]. For literature comparison purposes, we used CTV as the tumor volume (cm^3^).

RN can be seen between two to 23 months following radiation therapy [31]. RN was assessed subjectively using advanced MR imaging including diffusion-weighted imaging (DWI), apparent diffusion coefficient (ADC) mapping, MR spectroscopy, and PET. Factors suggestive of RN include 1) increased proportion of hypointensity on a T2-weighted MR image compared to the same area on a contrast-enhanced T1-weighted image and 2) any complete lack of perfusion, while ruling out the presence of a nodular highly vascularized area, within a contrast-enhanced lesion on perfusion MRI [31].  All patients suspicious for RN underwent MR spectroscopy and PET, where tumor progression was suspected for a standardized uptake value (SUV)/background threshold ratio > 1.5. Any toxicity not clearly due to tumor progression was deemed treatment-related toxicity.

Lesion stability on MRI was defined as the absence of new lesions or increased contrast enhancement > 25% in tumor size. Progression was defined as > 25% increase in tumor size. Patients were followed until death or until the censoring date (October 2015).

### Endpoints

The primary endpoint of this study was local (resection cavity) time to progression. Pre-HFSRT MRI was compared to post-HFSRT MRI to determine treatment response. Local control was defined as the absence of new nodular contrast enhancement > 5 mm from the resection cavity. Diffusion-weighted MRI ADC mapping and brain PET were used to differentiate between postoperative changes and tumor recurrence. Dexamethasone dependence at three months post-HFSRT treatment was recorded to compare to steroid status prior to HFSRT.

## Results

### Patient characteristics 

Twenty-one patients (16 men, 5 women) with histologically confirmed GBM were reviewed in this study (Table 1). The median age was 55 years (range: 26 – 74 years), and median KPS was 70 (range: 50 – 90). In accordance with the new recursive partitioning analysis (RPA) model described by Li, et al., the majority of patients in this study were in RPA Class IV (71.4%); four patients (19%) were in Class V, and two patients (9.5%) in Class III [32].

**Table 1:** Patient and Tumor Characteristics *Deep structures include: hippothalamus, splenium, perisylvian gyrus, cingulate gyrus, and corpus callosum **Based on RANO criteria [31].

Characteristic	No. (%)
Number of lesions treated	22
Gender	
Men	16 (76.2)
Women	5 (23.8)
Age (years)	
Median	55
Range	26 - 74
KPS	
< 70	4 (19.0)
³ 70	17 (81.0)
RPA Class	
III	2
IV	15
V	4
Site of recurrent lesion	
Temporal	2 (8.7)
Fontral	10 (43.5)
Parietal	4 (17.4)
Deep structures*	6 (26.1)
Time from primary radiotherapy (months)	
Median	9.6
Range	3.1 - 68.1
Treatment for primary GBM	
Radiotherapy	
Median dose (Gy)	60.0
Median number of fractions	30
Concurrent chemotherapy	
Temozolomide	21
Treatment for recurrent GBM	
Stereotactic radiotherapy	
Median dose (Gy)	33
Median number of fractions	3
Concurrent chemotherapy	
Bevacizumab	10
Temozolomide	3
Bevacizumab and temozolomide	1
Erlotinib	1
Pre-radiation size based on MRI (cm)	
Median	0.66
Range	0.13 - 4.08
Pre-radiation volume based on MRI (cm3)	
Median	1.07
Range	0.11 - 16.64

The median dose of EBRT used to treat the primary tumor was 60 Gy in 2 Gy fractions. The median time elapsed between initial EBRT and HFSRT was 9.6 months, where 18 patients (85.7%) had completed EBRT at least six months prior to HFSRT.

A diagnosis of GBM was made from surgical resection in 20 patients and via biopsy in one patient. Tumor localization was in the frontal lobe in 10 lesions (43.5%), in the parietal lobe in four lesions (17.4%), in the temporal lobe in two lesions (8.7%), and in deep brain structures in six lesions (26.1%). Median tumor size was 0.66 cm^2^ (range: 0.13 – 4.08 cm^2^). Median tumor volume was 1.07 cm^3^ (range: 0.11 – 16.64 cm^3^). 

### Local control

**Figure 1:**
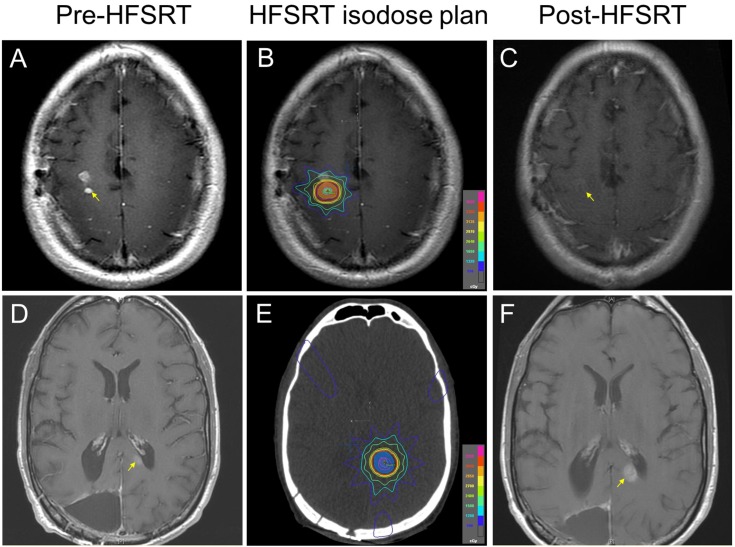
Representative images of MRI prior to and after therapy and radiation treatment plans of patients’ recurrent GBM tumors treated with stereotactic radiation (A) A right contrast-enhancing lesion in the superior centrum semiovale, visualized on a T1 contrast pre-treatment MRI scan (arrow). (B) A stereotactic radiation plan demonstrating isodose lines covering the target lesion. (C) A follow-up axial T1 contrast MRI scan demonstrating a resolution of the tumor on the at 22 months post-treatment (arrow). (D) A left contrast-enhancing lesion in the major forceps of the corpus callosum visualized on a T1 contrast pre-treatment MRI scan (arrow). (E) A stereotactic radiation plan demonstrating isodose lines covering the target lesion. (F) A follow-up axial T1 contrast MRI scan demonstrating tumor progression at 2.2 months post-treatment (arrow).

The median survival from completion of HFSRT was 9.3 months (range: 1.7 – 33.6 months); five patients were alive, two of whom were disease-free, and 16 patients were dead at the time of this review (Table 2). The median survival for lesions > 1 cm^2^ was 7.6 months (range: 1.7 – 16.0). There were two cases of local progression of disease within 5 mm of the resection cavity; one patient progressed at 2.2 months with a median survival of 5.9 months, and the other patient progressed at 9.6 months with a median survival of 10.0 months (Table 3). This yielded a median time to progression of 5.2 months. Both patients who progressed were receiving concomitant TMZ with HFSRT. Local control was achieved in 20/22 lesions (90.9%), where median survival was 9.3 months in these patients. Representative images of successful local control and progression are shown in Figure 1.

**Table 2:** Treatment Outcome of Stereotactic Irradiation of Recurrent GBM *Median survival from the time of HFSRT to death or last day of follow-up. **There were 13 patients who were receiving dexamethasone prior to HFSRT; decreased steroid dose was observed in 7/13 patients.

Treatment outcome	n = 22 lesions
Reoperation due to toxicity	0
Reoperation due to tumor progression	0
≥ Grade 2 toxicity	0
Local control (imaging response)	20 (90.1%)
Time to progression (months)	5.6
*Median survival (months)	9.3
**Decreased dexamethasone requirement	7 (54%)

**Table 3:** Lesion Stability after HFSRT *Time elapsed in months from pre-HSFRT to latest available MRI date. ^† ^% change in tumor size comparing pre- and post-HSFRT. ^‡^ Lesion stability on MRI was defined as the absence of new lesions or increased contrast enhancement > 25% in tumor size. Progression was defined as > 25% increase in tumor size.

Lesion	Pre-HSFRT size (cm^2^)	Time Elapsed (mo)*	Post-HFSRT Size (cm^2^)	% Change^†^	Lesion Stability^‡^
1	0.34	18.10	0.00	-100%	stable
2	0.48	3.60	0.37	-23%	stable
3	0.13	22.00	0.00	-100%	stable
4	1.43	4.60	0.77	-46%	stable
5	1.72	6.30	0.00	-100%	stable
6	1.39	3.30	0.25	-82%	stable
7	0.61	12.10	0.00	-100%	stable
8	2.27	1.70	1.38	-39%	stable
9	0.24	6.70	0.00	-100%	stable
10	1.32	10.70	0.91	-31%	stable
11	1.30	1.30	0.60	-54%	stable
12	4.08	5.00	0.00	-100%	stable
13	0.72	11.30	0.78	8%	stable
14	0.20	19.40	0.00	-100%	stable
15	0.16	9.60	0.25	56%	progressed
16	0.16	8.40	0.00	-100%	stable
17	1.08	8.40	0.00	-100%	stable
18	0.35	2.20	1.40	300%	progressed
19	2.99	4.20	1.98	-34%	stable
20	0.80	6.10	0.00	-100%	stable
21	0.18	5.60	0.00	-100%	stable
22	0.40	26.30	0.45	13%	stable

### Toxicity

No clinical signs suspicious for RN were reported. No patient had treatment-related neurological deterioration. There were eight patients who remained steroid-free both prior to and after HFSRT. Of the 13 patients who received dexamethasone prior to HFSRT, decreased steroid dependence was observed in seven patients (54%), where 4/7 of these patients (57%) eventually became steroid-free. Reoperation was not necessary for any patient. Fifteen of 21 patients (71.4%) received concurrent chemotherapy with HFSRT. Of these 15 patients, 10 received bevacizumab, three received TMZ, one received both bevacizumab and TMZ, and one received erlotinib due to positive EGFR status. There were no acute CTC/RTOG > Grade 2. All patients completed the course of HFSRT with no interruptions.

## Discussion

A conventional regimen of 2.0-Gy fractions to dose escalations of 90 Gy by three-dimensional conformal radiation therapy has been reported to confer better local tumor control [33]. However, a survival benefit has not been demonstrated in similar regimens of dose escalation to 90 Gy [34] or 84 Gy [35], and thus, hyperfractionated regimens remain controversial in their efficacy. The rapid doubling time inherent to GBM likely contributes to the ability of these tumors to repopulate in a short time. Indeed, it has been noted that approximately 19% of patients may have clinically progressed while undergoing conventionally fractionated radiation therapy [36].

In contrast, hypofractionation is able to deliver a higher biologically effective dose over a shorter treatment period and its advantages are two-fold. First, a higher dose per fraction can achieve increased cell damage. Second, by extension of decreasing the initial tumor burden, this would reduce the exponential tumor repopulation during the course of treatment. Furthermore, a shortened treatment duration could potentially lower the cost of treatment by decreasing costs associated with per fraction treatments, as well as decrease the number of trips by the patient and associated transportation costs. To date, the use of 11-Gy per fraction in this review is the highest dose per fraction reported. Yet, in the present study, patients diagnosed with small recurrent GBM treated with this hypofractionated regimen achieved a local control of 90.9%.

The toxicity profile in HFSRT regimens is varied (Table 4). The incidence of radiation damage reported in studies has ranged from 7% - 36%. The highest incidence (36%) was reported by the Royal Marsden experience, where 36 patients with recurrent GBM (median tumor volume 24 cm^3^) were treated with doses ranging from 20 – 50 Gy in 5-Gy per fraction [17]. Following a fractionation scheme of 25 Gy/5 fractions, Ciammella, et al. [11] reported neurological deterioration reversible with dexamethasone in 13% of patients. Another study using a median dose of 37.5 Gy/15 fractions in 25 patients reported clinical necrosis in one patient and cranial nerve palsy in another patient [10].

**Table 4:** Studies of Stereotactic Reirradiation for Patients with Recurrent GBM TMZ = temozolomide, TTP = time to progression (months) ^a^Bevacizumab, TMZ, irinotecan, bortezomib, epothiolne, sunitinib, sorafenib, vincristine, carboplatin ^b^Bevacizumab, TMZ, erlotinib *If the study had mixed Grade III and Grade IV gliomas, the number of Grade IV glioma is in brackets. ^**^Prospective study. ^†^Median dose/dose per fraction. ^‡^Time interval between treatment of primary GBM to HFSRT treatment of recurrent GBM (months). ^§^Toxicities based on CTC/RTOG. ^¥^Only PTV was reported.

First Author	n*	Concomitant Chemotherapy	Dose/Dose per fx (Gy)^†^	Interval to Recurrence (m)^‡^	Grade 3/4 Toxicities^§^	Median Tumor Size (cm^3^)	Local Control, Median Survival After HFSRT
Fokas [13]	53	none	30/3	12	0%	35^¥^	--
Vordermark [18]	19 (14)	none	30/5	19	0%	15^¥^	--
Dincoglan [12]	28	none	25/5	11.2	0%	--	--
Patel [16]	10	none	36/6	14.9	0%	51.1	50%, 7.6m
Ciammella [11]	15	none	25/5	10.8	13%	--	--
Hudes** [15]	19	none	30/3-3.5	3.1	0%	12.66	79%, 10.5m
Cho [10]	25 (15)	none	37.5/2.5	19	8%	25	44%, 12m
Fogh [19]	147 (105)	various^a^	35/3.5	8	0%	22	60%, 11m
Minniti [22]	36	TMZ	37.5/2.5	14	8%	13.1	--
Minniti [23]	54 (38)	TMZ	30/6	15.5	7%	9.8	--
Lederman** [21]	88	paclitaxel	24/6	6.3	10%	32.7	40%, 7m
Wurm** [24]	25 (20)	topotecan	25-30/5-6	12.8	0%	16.5	--
Greenspoon** [20]	31	TMZ	25-35/5	>6	13%	12	--
Current study	22	various^b^	33/11	9.6	0%	1.07	91%, 9.3m

A higher incidence of toxicities is seen in the setting of HFSRT with concomitant chemotherapy. Minniti, et al. reported RN-associated neurological deterioration in 8% [22] and 7% [23] of patients when treated with TMZ concurrent with 37.5 Gy/15 fractions and 30 Gy/5 fractions, respectively. In a prospective analysis, Lederman, et al. [21] documented RN in 10% of patients treated with 24 Gy/8 fractions and paclitaxel. Another prospective trial using a dose escalation of 25-35 Gy in 5-Gy fractions with concomitant TMZ reported RN in 13% of patients [20]. In the present study, 15/21 patients (71.4%) received chemotherapy tailored to their condition while undergoing HFSRT, and there were no incidences of RN or CTC/RTOG toxicities > Grade 2. While the use of bevacizumab has been reported to suppress RN [37], 10/21 patients in the present series did not receive bevacizumab. Furthermore, of the patients dependent on steroid use prior to HFSRT, 54% experienced a decrease in steroid dose after HFSRT. This is comparable to a 60% decrease in steroid dose observed in a Phase I dose escalation study of 19 patients treated with 30 Gy in 3-3.5 Gy fractions [15]. Lastly, a short follow-up duration could account for the lack of RN observed. However, median survival time amongst the five survivors in the present study was 23.3 months, which falls within the 2 – 23 months period where RN is most likely observed [31]. For instance, Floyd, et al. [38] reported survival of patients with brain necrosis at 23, 20, and nine months.

Our results show that HFSRT delivered in three fractions of 11-Gy each is well tolerated with no significant acute side effects. This is likely due to the small size of the tumor, reducing the volume of the normal brain receiving radiation. Hudes, et al. [15] demonstrated that a tumor volume < 20 cm^3^ showed a greater probability of treatment response, and the maximum volume in our study was 16.64 cm^3^. Greenspoon, et al. [20] demonstrated increased efficacy in the treatment response for tumors < 3 cm in diameter. The median diameter of tumors irradiated in the present study was 0.91 cm (range: 0.4 – 2.4 cm).

There is a paucity of data in examining local control rates and time to progression of recurrent GBM tumors. Literature reports a local control rate between 44% - 79% [10, 15-16, 19, 21]. A local tumor control of 90.9% is the highest reported so far. However, different methods of documenting tumor status make comparisons across studies difficult. For instance, Fogh, et al. [19] reported stable disease in 60% of patients based on the three-month follow-up MRI scan. The present study uses the last available follow-up MRI scan to determine disease state.

While this was a retrospective study and patient selection has been biased, the majority of patients were in RPA Class IV and V. A German study that followed 492 patients diagnosed with primary GBM over the course of 17 years reported a median age at diagnosis of 62 years [39]. An age > 50 years stratifies patients into RPA Class IV and V [32]. Therefore, the patient population in the present study, 90% of which are Class IV and V, is representative of the GBM population at large. Due to the stringent selection bias of small recurrent tumors, there was no control arm using the conventional fractionation scheme to compare to. In view of the high dose per fraction used, the authors felt that it was important to first establish the safety and feasibility of such a regimen at our institution. Due to the small patient sampling size and lack of control arm, pursuing overall survival values would not have been statistically meaningful. Furthermore, given that treatment of recurrent GBM is palliative in nature, the focus of this study is to examine local control and incidence of RN rather than the impact on overall survival.

## Conclusions

The current study is the first to use very high-dose/fraction (11-Gy/fraction) in the treatment of GBM. Our study confirms the feasibility and safety of a hypofractionated regimen of 33 Gy/3 fractions in achieving local control with no radiation necrosis in patients with recurrent small GBM and provides support to conduct prospective and randomized trials to validate these results.
